# How to Interpret a Nerve Conduction Study: A Stepwise Approach

**DOI:** 10.1212/NE9.0000000000200275

**Published:** 2025-11-13

**Authors:** Aaron S. Zelikovich, Gabriela Figueiredo Pucci, Jonathan Minkis, Marcus V. Pinto

**Affiliations:** 1 Department of Neurology, Lenox Hill Hospital, Northwell Health, New York, NY;; 2 Department of Neurology, University of Pittsburgh, PA; and; 3 Department of Neurology, Mayo Clinic, Rochester, MN.

Nerve conduction studies (NCS) assist in the diagnosis and characterization of neuropathy and differentiate between axonal and demyelinating forms.^1^ Interpreting NCS can be challenging for neurology trainees.^2^ We highlight 4 key components of interpreting basic NCS in clinical settings (Figure).

**Figure:**
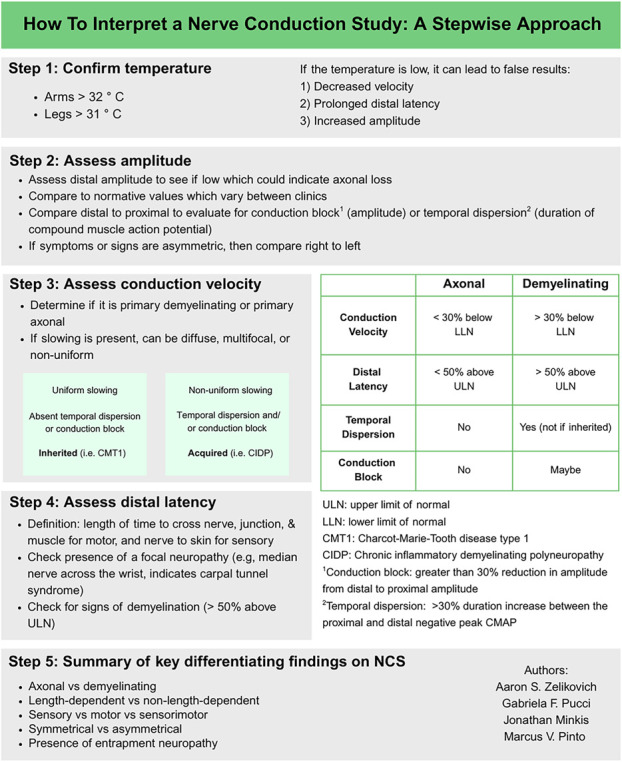
A Stepwise Approach to Interpret a Nerve Conduction Study

The 4 steps in interpreting NCS are: confirming temperature, assessing amplitude, conduction velocity, and distal latency. Cool temperatures can lead to false-positive studies. Amplitude and velocity help to differentiate between primary axonal and demyelinating forms of neuropathy. Distal latency helps assess for slowing across short nerve segments, what is important in the evaluation of focal neuropathies and demyelinating polyneuropathies.
